# Poster 1001: ALLERGENIUS®: an expert system for the interpretation of allergen microarrays

**DOI:** 10.1186/1939-4551-7-S1-P2

**Published:** 2014-02-03

**Authors:** G Walter Canonica, Giovanni Passalacqua, Gianni Melioli

**Affiliations:** 1 Genoa University, Dept Of Internal Medicine Allergy and Respiratory Diseases, Genoa, Italy; 2 Dept of Internal Medicine, Genoa University Allergy and Respiratory Diseases, Genova, Italy

## Background

The introduction of a microarray containing 112 different components in the management of allergic patients has significantly modified the diagnosis and the treatment in polysensitized patients (Sastre, 2012, Passalacqua, 2013), because of the large number of added values generated by this approach in comparison with other diagnostics based on extractive – non purified –allergens. However, microarray interpretation is sometimes challenging, for the high number of possible combinations of 112 different allergens, the very large number of interactions between different components, the presence of either genuine or cross-reacting components, the presence of both inhalant and food allergens (Melioli e Canonica, 2012).

## Methods

An expert system, based on Flex, a language related to Prolog, has been developed. The characteristics of the different components and the rules governing the relationships between allergens were obtained from medical literature and specialized web sites. The inputs of the expert system are the age and the diseases of the patient, Skin Prick Tests and specific IgE results (when available) and microarray (ISAC) results.

## Results

The expert system output is represented by a text file containing patient’s identification and the description of the general situation (mono or poly-sensitization, cross- or co-sensitization). The patient’s phenotype (Melioli, 2013) and its relationship with expected immunotherapy results (Schmid-Grendelmeier, 2010) are also shown. A list of warnings, related to a sensitization to potentially dangerous components (such as LTPs, Peanuts etc.) is presented. At the end of the introduction, all positive components (inhalants, food, latex and venoms) are listed and commented. Then, families of positive cross-reacting components (such as PR-10, profilins, tropomyosins etc) are discussed. A special section (termed “post molecular anamnesis”) suggest the doctor any clinical questions that should arise from the microarray interpretation (such as the mite-shrimps syndrome in the presence of a sensitization to Der p 10). Finally, two other sections (the first focused on therapeutic suggestions for inhalant allergy and the second of possible discrepancies between SPT /sIgE and microarray results) are available. Of note, the final report has hyperlinks to a dedicated web site to add further information about allergens, component, diseases etc. The “in silico” validation of the expert system (Allergenius v4.2) was successful and the clinical validation in progress.

## Conclusions

The potential impact of this technology in the management of complex polysensitized patients, namely a more proper prescription of Allergen Immunotherapy, could be really relevant.

